# The Viscoelastic Properties of Passive Eye Muscle in Primates. I: Static Forces and Step Responses

**DOI:** 10.1371/journal.pone.0004850

**Published:** 2009-04-01

**Authors:** Christian Quaia, Howard S. Ying, Altah M. Nichols, Lance M. Optican

**Affiliations:** 1 Laboratory of Sensorimotor Research, National Eye Institute, Bethesda, Maryland, United States of America; 2 Department of Neurology, The Johns Hopkins Hospital, Baltimore, Maryland, United States of America; Universidad Europea de Madrid, Spain

## Abstract

The viscoelastic properties of passive eye muscles are prime determinants of the deficits observed following eye muscle paralysis, the root cause of several types of strabismus. Our limited knowledge about such properties is hindering the ability of eye plant models to assist in formulating a patient's diagnosis and prognosis. To investigate these properties we conducted an extensive *in vivo* study of the mechanics of passive eye muscles in deeply anesthetized and paralyzed monkeys. We describe here the static length-tension relationship and the transient forces elicited by small step-like elongations. We found that the static force increases nonlinearly with length, as previously shown. As expected, an elongation step induces a fast rise in force, followed by a prolonged decay. The time course of the decay is however considerably more complex than previously thought, indicating the presence of several relaxation processes, with time constants ranging from 1 ms to at least 40 s. The mechanical properties of passive eye muscles are thus similar to those of many other biological passive tissues. Eye plant models, which for lack of data had to rely on (erroneous) assumptions, will have to be updated to incorporate these properties.

## Introduction

Recent studies of monkeys with one eye muscle paralyzed [1], [2] have revealed an intricate pattern of static and dynamic deficits, which cannot be fully reproduced using current models of the eye plant (the globe, extraocular muscles and passive tissues in the orbit). We have argued elsewhere [3] that the inability to capture the static deficits is probably due to our limited knowledge of the innervation patterns in physiologic conditions. However, the failure to capture the dynamic deficits must almost certainly be ascribed to our limited knowledge about the dynamic mechanical properties of eye muscles, especially in their passive state.

Because no complete review of the pertinent literature is available, we will now briefly summarize what has been reported regarding the passive properties of eye muscles.

### Static forces

The relationship between the length of an eye muscle and the force it generates at equilibrium (i.e., after the length has been maintained for a very long time) has been studied in several species. Robinson [4] and Collins [5] were first, and used cats. Collins proposed that the static stiffness of the muscle (which he defined as the slope of the length-tension relationship) was proportional to the force at the same length. That is, Collins implicitly proposed that the stiffness and the force both increase exponentially with length. In his original paper, results from two experiments were plotted. We found that one dataset (his Fig. 15) can indeed be fit very well (r^2^ = 0.99) by the following exponential function:
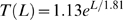
where *T* is the tension (in gf) and *L* is the elongation (in mm) relative to the muscle length with the eye in primary position. The other dataset (his Fig. 8) is less well captured (r^2^ = 0.89) by a single exponential, but is considerably better fit (r^2^ = 0.97) by the following expression:
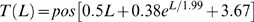
where *pos[ ]* indicates that negative values are truncated to zero.

**Figure 1:**
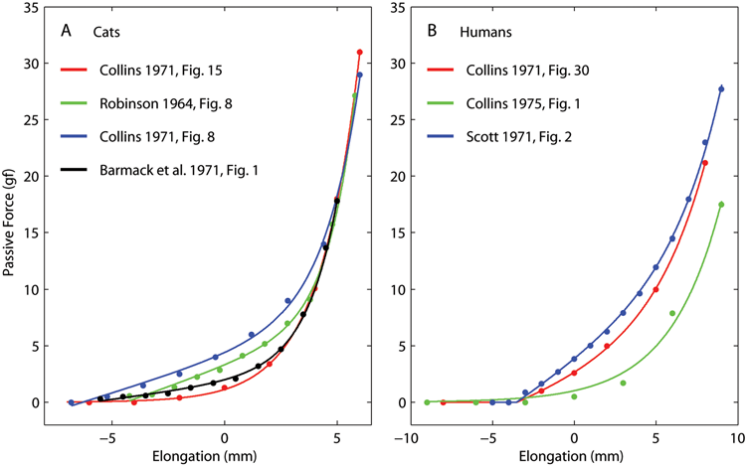
Passive force-length relationships reported in the literature. A: Data from the lateral rectus in cats, pooled across studies. The red fit represents original data, whereas the other fits have been scaled along both axes (see text) in an (obviously failed) attempt to reconcile the various data sets. B: The passive force-length relationship in human horizontal recti, as measured in studies on strabismic subjects (see text). The elongation is referred to the straight ahead position.

Obviously, in this latter expression the stiffness (i.e.,, the derivative of the force with respect to length) would not be directly proportional to the force. However, we found that, unlike the pure exponential proposed by Collins, it provides a good fit to all the datasets available in the literature. For example, the passive length-tension curves in cat extraocular muscles (EOMs) were measured in two other experiments. The data reported by Robinson [4] is well fit (r^2^ = 0.96) by
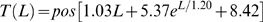
In this case however, the elongation (*L*) is relative to the length at which the active force in the tetanized muscle is maximal. Robinson assumed that this was equal to the length in primary position, but we now know that that is not true. The data collected by Barmack and colleagues, also in cats, [6] is well fit (r^2^ = 0.97) by:
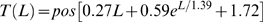
where *L* indicates the elongation relative to the length in primary position. In each study a different value was chosen for the zero length, and there must be some variability across animals, thus it is not surprising that the various fits look quite different. In an effort to find some sort of average fit, we tried to shift and scale the length axis, and to scale the force axis. Using reasonable ranges (±5 mm shifts and ±30% scaling), we could not find decent agreement across all curves (Fig. 1A, see legend).

Unfortunately, there are not many studies in other species. Barmack [7] studied EOM passive force in rabbits, and found that the length-tension relationship in their inferior rectus is almost linear. We could fit it very well (r^2^ = 0.96) with:
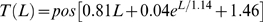
In this case *L* indicated the elongation relative to primary position, but Barmack noted that the precision of this measurement was quite low. The only study in monkeys [8] produced results that are somewhat different from those in all other studies. The force is low for the first four mm, and does not even seem monotonic over this range. It then increases linearly first (over four mm) and very rapidly afterwards (over two mm). Excluding the maximum elongation point (which is most likely beyond the muscle's natural working range) from their dataset, we obtained a reasonably good, but not great, fit (r^2^ = 0.92) with:
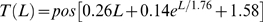
Two other animal studies exist, but their methodologies make them uninformative: Stone and colleagues [9] measured the force on the globe with muscles attached in dogs, and Breinin [10] provided only relative forces in cats.

Several studies have been published in which the passive force was measured in humans before strabismus surgery (Fig. 1B). The first two such studies [5], [11] both report the same data from a patient in which the passive force is estimated by having the awake patient fixate (with the other eye) as far as possible away from the muscle's field of action. Obviously this is not an ideal experimental condition, and most likely the muscle was somewhat innervated. The length-tension curve is fit perfectly (r^2^ = 0.99) by:
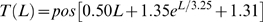
A later study [12] found, using the same methodology, somewhat lower forces, which are reasonably fit (r^2^ = 0.90) by
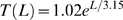
Adding parameters to this fit did not improve it significantly. In yet another study [13], the passive forces from five deeply anesthetized patients from the same group were reported. In this case a good fit (r^2^ = 0.97) is obtained with:
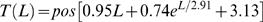
The data from humans, having been collected by the same group of investigators, and thus using more standardized techniques and equipment, should be considerably more consistent than those in cats. However, this expectation is not met (Fig. 1B). Also, because of the different methodology used, one would have expected the force measured by Scott (which should also be more reliable, being the average of five subjects) to be lower than the force reported in the other two studies, but this was not the case.

### Dynamic forces

There is only one applicable study of the dynamic properties of passive eye muscles [5], on the lateral rectus of cats. Collins concluded that the passive muscle can be modeled with an elastic element in series with a Voigt element (a viscous and an elastic element in parallel); the (only) time constant in the model was 100 ms. (Another study was carried out in dogs, but the force was measured at the eyeball with an intact plant [9]; parsing the contributions of the muscles and orbital tissues under these circumstances is a hopeless endeavor.) This result would seem to indicate that passive eye muscles have little in common with any other passive biological tissue studied.

This brief but complete review of the literature reveals a striking paucity of data and considerable inconsistency across data sets, even within the same species. Even more worrisome, there are considerable inconsistencies even across subjects within the same experimental group. Building models based on such limited knowledge is obviously less than ideal. Robinson recognized this problem almost 30 years ago [14]. Referring to the viscous properties of passive eye muscles, he noted that “there seems little doubt that the value chosen for this viscosity will have a considerable effect on a model's behavior and our lack of any certain knowledge of its properties is certainly a source of indeterminacy in any model.” Robinson continued: “I believe we are now at the point where we need more facts about how muscles behave, rather than more modeling”. Unfortunately, since that landmark review article was published not a single experiment has been carried out to measure the dynamic mechanical properties of passive eye muscles.

In an attempt to fill this glaring gap, we measured, *in vivo*, the forces exerted by passive extraocular muscles of monkeys. While the lion's share of this study is devoted to the dynamic properties of passive eye muscles, in this article we will also present a detailed analysis of the static forces.

## Methods

### Ethics Statement

All procedures were in agreement with the Public Health Service policy on the humane care and use of laboratory animals and all protocols were approved by the Animal Care and Use Committee of the National Eye Institute.

### Animals

Eye muscle forces were measured in three adult rhesus monkeys (*Macaca mulatta*), ranging in weight from 8 to 14 Kg (identified as m2, m3, and m4). None of the animals had been previously used in any experiment, and their eyes and orbits were thus pristine. They had all been exposed to the simian herpes B virus, and accordingly were isolated and considered inappropriate for awake, chronic experiments.

### Surgical procedure

The animal was premedicated with ketamine hydrochloride (10 mg/Kg) and glycopyrrolate (13–17 µg/Kg) delivered intramuscularly. An IV catheter was placed in the saphenous vein, and lactated Ringer's solution was administered (10 mL/Kg/hour). The animal was then placed supine on the surgical table, intubated and anesthetized with isoflurane (2–4%) in oxygen, and mechanically ventilated. Heart rate, indirect mean arterial blood pressure, mucus membrane color, peripheral oxygenation/SpO_2_, end-expiratory CO_2_ partial pressure, and EKG were monitored and maintained within normal physiological ranges. Body temperature was monitored and maintained at 37°C with a heating pad. Paralysis was induced with pancuronium bromide (0.05–0.10 mg/Kg IV), and maintained with 0.025–0.050 mg/Kg IV every 45 minutes until the end of the procedure. The paralytic agent was used to ensure that the muscles were completely passive. It is usually assumed that deep anesthesia is sufficient to obliterate muscle activation, but only a paralytic agent can guarantee this outcome. Pancuronium bromide, a non-depolarizing agent, is the preferred agent; succinylcholine, another commonly used paralytic, could not be used here, as it actually activates an entire class (multiply innervated, non-twitch) of eye muscle fibers [15]. After measurements were finished, the animal, without awakening, was euthanized with an overdose of sodium pentobarbital (150–250 mg/kg). The animal was then perfused intracardially with a glutaraldehyde and paraformaldehyde solution. The orbital contents were preserved for anatomical study.

### Experimental procedure

After the animal had been anesthetized, its head was stabilized with a stereotaxic device's ear bars (to reduce the head's degrees of freedom from six to one). A mouth bar added to the stereotaxic device was attached to the front teeth with dental cement to fix the head so that Reid's baseline was perpendicular to the table. Both eyes were prepped and draped in the usual sterile ophthalmic manner. The conjunctiva was then incised in correspondence with an eye muscle insertion on the globe, and a muscle hook was placed under the insertion. From here we adopted two different techniques.

In four muscles (identified as m2LR, m2SR, m3LR and m3SR), the muscle was connected to the measuring device directly by a Kevlar™ thread (between 50 and 75 mm long). The connection was achieved by sandwiching the wire, together with the tendon, between two tiny titanium plates (6 mm by 2 mm by 1 mm) kept together by two microscrews (total weight 0.05 g). The pressure exerted by the screws was such that no slippage could have occurred. To err on the side of caution, a small knot (fixed with glue) was placed at the distal end of the Kevlar wire, and before tightening the screws the wire was pulled until the knot came in contact with the distal side of the plates. The proximal end of the Kevlar wire was connected to the measuring apparatus by a knot secured with a drop of cyanoacrylate glue. The Kevlar 49 thread we used (0.2 mm diameter, 460 denier (d)) has a tensile modulus of 885 g/d (Dupont Kevlar Technical guide, see Dupont web site). The stiffness of the connection was then between 5400 and 8100 gf/mm.

On the last muscle tested (identified as m4LR), we did not use the above described clamping technique, but instead tied a Surgidac™ (US Surgical) 5-0 surgical suture to the tendon and then knotted its other end to the distal end of the Kevlar wire (the knot was then secured with a very small metallic crimp, weight 0.02 g). More precisely, the suture was tied at both sides of the tendon, and both ends of the suture were then connected to the Kevlar wire (i.e., it was as if there were two sutures connected in parallel). The length of the suture segment was 8 mm, whereas the length of the Kevlar segment was 50 mm. We selected Surgidac sutures because they are the least compliant of those we tested (the others were Ticron™, Fiber-wire™, Tenara™, Gore-tex™, Vicryl™ coated and uncoated, Dexon™, and braided silk, listed in order of ascending compliance). A double 8 mm segment has a stiffness of 3520 gf/mm. The overall stiffness of the Surgidac-Kevlar connection was then 2450 gf/mm.

In all cases the tendon was connected to the measuring device before being detached from the globe, allowing us to get an estimate of the force exerted by the muscles when the line of sight pointed the equivalent of straight ahead (i.e., in this posture, straight up). Since the animal was anesthetized, straight ahead position was estimated by the Hirschberg corneal reflex test, which has an approximate accuracy of five degrees horizontally and vertically.

Muscle force was measured using an Aurora Scientific (Aurora, ON, Canada) 305C Dual-Mode Muscle Lever System. In the experiments described here we imposed the muscle length, and measured the corresponding change in force (NB: the SI standard unit of force is the Newton (N), but muscle force is traditionally measured in units of *gram force* (1 gf≈0.0098 N); e.g., a mass of 102 g exerts a force of 102 gf, or 1 N, on earth). The specifications for the system used are as follows:

- Length Signal Resolution: 1 micron
- Length Signal Linearity: 0.1% over the center 4 millimeters, 0.5% over the entire 20 mm range
- Length Step Response Time (1% to 99% critically damped): 2.0 msec
- Sinusoidal Frequency Response (−3 dB): 330 Hz
- Force Signal Resolution: 1.0 mN (∼0.1 gf)
- Force Signal Linearity: 0.2% of force change

Both the length and the force signals are low-pass filtered with a 4^th^ order Butterworth filter with a cut off frequency of 5 kHz. The bandwidth of the system is limited by the motion bandwidth, not by the sensor bandwidth. In all our experiments we stayed well within the bandwidth of the equipment. In doing so we guaranteed that the measurement device was not a limiting factor, and that both the length and force sensor outputs can be treated as veridical. The input/output analog signals for/from this device were generated and acquired through an A/D-D/A interface board (National Instruments, NI USB-6211) connected to a laptop PC (IBM, Amonk, NY) and controlled by LabView (National Instruments, Austin, TX). The experiment was controlled by a custom Java program that communicated with LabView, displayed the data in real-time, and stored it for later analysis.

Sterile artificial tears were used to bathe the exposed tissues continuously during the experiment. Before recording we preconditioned the muscles by repeatedly (5–10 times) stretching and releasing them sinusoidally over their entire range (which is standard procedure in tissue rheology to guarantee repeatable results; the relatively low number of cycles used here is justified by the *in vivo* condition, which is unique to our experiment). We were extremely careful to preserve the blood supply and to keep the tissues well hydrated, because it has been recently shown that other methods (e.g., extraction of the muscle) are fraught with potential problems [16]. For all muscles tested, we ran a block of 3–4 ramps at the beginning and end of the experiment to test for any possible deterioration of the muscle. We did not observed any significant change in these test trials.

Because very little was known about the viscoelastic properties of passive eye muscles (and, as we show here, that little turned out to be grossly inaccurate), we based our experimental design on the results and modeling studies from other passive biological tissues. We concluded that the best experimental design to characterize the *in-vivo* viscoelastic properties of eye muscle consists in imposing small elongation steps, executed within a few milliseconds, from initial lengths spanning the entire elongation range tested. All the steps we imposed had an amplitude of 0.5 mm. In all muscles we used steps that had a peak speed of 160 mm/s, a peak acceleration/deceleration of 144 mm/s^2^, and a duration of 4.5 ms (bandwidth 130 Hz, Welch's method). In some muscles we also induced some slower steps, with a peak speed of 80 mm/s, a peak acceleration/deceleration of 74 mm/ s^2^, and a duration of 8 ms (bandwidth 50 Hz). Long waiting periods were imposed before and after each length change. Other paradigms (e.g., constant-speed ramps spanning the entire elongation range, at various speeds: 1, 10, 80, and 160 mm/s) were also part of the experiments, but they will be described and analyzed in subsequent papers.

Because it was technically impossible for us to measure the forces during shortening (they become negative for even relatively low shortening speeds, causing the Kevlar thread to buckle) only lengthening was tested. Knowing the passive properties during shortening would be valuable to study pathologic conditions, but a completely different measuring apparatus would have to be constructed. Fortunately, during shortening in physiological conditions the muscle is always innervated, and so its passive properties are less important. Another limitation of our study is that, because after each muscle elongation we waited for a long time (30 seconds in the first two monkeys, 45 s in the third) for the force to settle, we could not perform all experiments in all muscles (we never exceeded a one-hour testing period per muscle, as we wanted to avoid any tissue deterioration).

The elongation range was determined separately for each muscle. As a lower bound we picked the longest muscle length at which the force recorded was essentially zero. This length coincided with, or was very close to, our approximate estimate of the muscle length with the eye in primary position. For the upper bound we selected the length at which the force curve steepened to the point where elongations of a tenth of a millimeter caused considerable force changes (around 1 gf). To avoid any damage, we never pulled the muscle further, even though it was clearly possible to do so; we are confident that the range tested always covered the entire oculomotor range (i.e., the set of lengths that are achieved in physiologic conditions, which in monkeys correspond to approximately 45° of rotation), but never exceeded it by more than one mm. Accordingly, the elongation range tested was always about eight mm. We never noticed the sudden increase in stiffness corresponding to the leash region described by others [17]. On a couple of occasions, after testing was completed, we slowly stretched the muscle by an extra two mm, but even then no sudden stiffening was noticed.

We originally considered running multiple trials for each condition, with the intention of increasing the signal to noise ratio by averaging across the trials. As soon as we started the experiments, we realized that most of the noise we observed was not independent and randomly distributed, which could be reduced by averaging over trials. Rather, we measured significant heartbeat and respiration-related signals, neither of which would go away with *small n* averaging. The actual measurement noise was extremely small, at or below the level of our instrumentation accuracy. Accordingly, we collected a single trial per condition.

Another aspect that became clear early on was that the muscle needed to be completely detached from the globe before the measurements. When we prepared our very first muscle for measurement we were careful to be minimally invasive, detaching only the tendon and immediately starting the measurements. We quickly realized that, because of other attachments between the muscle and the sclera, as we pulled the muscle the eye rotated with it. Also, connective tissues on the orbital side of the muscle were dragged out of the orbit at longer extensions. Evidence for the mechanical significance of these extra-tendinous attachments in humans has been recently reported [18]. From then on, before starting the measurements, we carefully “cleaned” the muscle, detaching all the connections between the global side of the muscle and the sclera, and the most distal attachments between the orbital side and the bony orbit.

### Noise

In all our measurements we could clearly identify three sources of noise:

- A high-frequency, low-amplitude signal that we refer to as measurement noise, meaning that it is probably not part of the muscle force, but is rather due to our recording system.
- A physiological signal with a base frequency of approximately 1.5–1.6 Hz, corresponding to the heartbeat.
- A physiological signal with a base frequency of approximately 0.4 Hz, corresponding to the ventilation rate.

Two sources of measurement noise could be identified in the frequency domain. The first one covers a band between 2 and 11 Hz, with a dominant frequency around 5 Hz, and contains 75% of the total power of the high-frequency noise. This component is most likely due to micro-oscillations of the Kevlar suture connecting the muscle tendon to the apparatus. The second source of noise is the AC power line, with most of the noise (6% of the total power) scattered across the first (60 Hz) and third (180 Hz) harmonics. The RMS force of the high-frequency noise was always very small, below the accuracy of our instrumentation (0.1 gf).

### Denoising

The two physiological noise sources were stronger than the measurement noise, especially the heartbeat noise, and increased approximately linearly with the stiffness of the muscle (which, as we'll see below, increases with muscle length). We estimate that periodic changes in muscle length of the order of ±10 µm would be sufficient to induce the noise we measured. The physiologic noise could not be removed by frequency-band filtering, because the frequency spectrum of the noise overlapped the spectrum of the signal during the early part of the post-elongation force decay. To overcome this problem we took advantage of two properties of our measures: 1) the noise waveform is highly consistent, and 2) the force decays exponentially, so that most of the high frequency components of the signal (dominated by short time constants) is clustered during and just after the elongation period. We thus proceeded as follows. First, we fitted all the post-movement traces with a sum of exponentials using the Emri-Tschoegl algorithm (E-T), described below. Starting from 5 s after the end of the elongation phase, this fit to the raw data was insensitive to the biological noise. Accordingly, we used the residuals (i.e., the difference between the measured force and the fit) from the slow part of the decay curve to form templates accurately describing the physiological noise.

**Figure 2:**
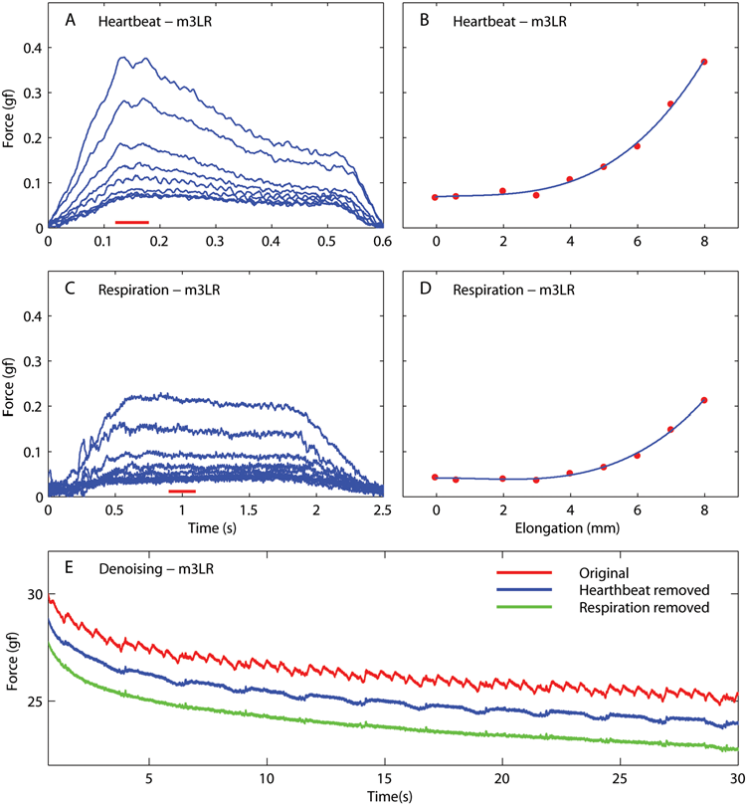
Sources of noise in our force measurements. Data are from the lateral rectus (LR) of the second monkey (m3). A: Heartbeat noise templates at different muscle lengths. B: Magnitude of the heartbeat noise (average over the time interval indicated by the red bar in A) as a function of muscle length. A least-squares cubic fit to the data is shown in blue. C: Respiration noise templates at different muscle lengths. D: Magnitude of the respiration noise (average in the time interval indicated by the red bar in C) as a function of muscle length. A least-squares cubic fit to the data is shown in blue. E: Red: Part of the relaxation response measured in the same muscle after a quick step. Blue: Same as red trace, but after template-based removal of the heartbeat noise (shifted down by 1 gf for clarity). Green: same as blue trace, but after template-based removal of the respiration noise (shifted down by 2 gf for clarity). The green trace is the denoised data used in all subsequent analyses.

We first used these residuals to build a template for the heartbeat noise (by averaging over many heartbeat cycles). This was done separately at each muscle length sampled. In Fig. 2A we show the heartbeat templates for one muscle (lateral rectus in m3); each trace corresponds to a different muscle length. In Fig. 2B we show the relationship between the muscle length and the magnitude of the heartbeat noise, measured around the peak (red bar in Fig. 2A). Each red dot in Fig. 2B corresponds to a different trace in Fig. 2A, and a cubic fit through the data is shown. Next, we subtracted this average template from the residuals. We then assumed that the periodic waveform that was left was due to the respiration noise, and thus collected and averaged those cycles to compute, for each muscle length, a template of the respiration noise. In Fig. 2C we show the respiration noise templates, and in Fig. 2D we show how their magnitude varies with muscle length. These two sets of templates were then, in a semi-automatic way, matched to the recorded force (taking into account the instantaneous muscle length to interpolate across noise templates), and the two sources of noise were then subtracted off sequentially. In Fig. 2E we show an example of the results. The red trace is the original post-step decay, the blue trace is what is left (shifted down for clarity) after the heartbeat noise is removed, and the green trace is what is left (again shifted down) after the respiration noise has also been subtracted. Obviously the result, which is representative of what we got on all our traces, is very good, leaving behind only the high frequency, low amplitude, measurement noise described above. It should be pointed out that our noise templates do not have zero mean, as we assume that the biological sources of noise increase the force measured. The E-T fit was then recomputed from the denoised data set; only the fits to denoised data are included in the Results.

## Results

### Static length-tension relationship

The static length-tension relationship of a viscoelastic material describes the steady-state force exerted at a given length (it is the equivalent of the linear equilibrium stress-strain law of infinitesimal elasticity theory [19]). Obviously, it cannot be directly measured, as at each length the force would require an infinite amount of time to reach steady-state. Practically, the static force at a given length can be estimated from force measurements in two ways. First, one could simply record the force measured after the muscle length has been kept constant for at least three times the longest time-constant of interest of the system. It also would be advisable to reach the final length with as slow a movement as possible, because, other things being equal, one would assume that the larger the viscous force induced by the preceding movement the larger the error in the estimate. Alternatively, one could use a model to extrapolate the asymptotic force at which the muscle would eventually settle. In this case, the estimate can be as accurate as the model used to fit the response. We used a hybrid approach to estimate this relationship. We estimated the force at the length following each quick step using the asymptotic value from the spectral fit (described in the Relaxation Response section). As an estimate of the force at the length preceding each quick-step we instead used the average force measured during 200 ms before the step (after denoising the signal, see Methods). As the spectral fits were always extremely good, we believe that the former estimates are highly reliable. The latter were of course less reliable, somewhat underestimating the force exerted at the shortest length (because the force was still recovering after the shortening), and overestimating the force exerted at the other lengths (because the force was still settling after the previous lengthening). As the preceding fast movement was always at least one minute away, we believe that these estimation errors were quite small.

We evaluated in this manner the length-tension relationship in five recti muscles (three lateral recti, two superior recti) in three monkeys. After failing to fit the static data with previously proposed equations (e.g., [5], [20], [21]), we settled on the following relationship:
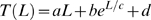
(1)where *T* is the passive force, *L* is the muscle elongation, and *a*, *b*, *c*, and *d* are parameters. We used a weighted sum-of-squares minimization procedure because of the different reliability associated with various data points (see above). The distance between each data point and the fit (i.e., each residual) was weighted as follows: residuals for the asymptotic values have a unitary weight, positive residuals for the pre-step averages had a weight of 0.1 for the first step and 0.5 for all others, and negative residuals for the pre-step averages had a weight of 0.5 for the first step and 0.1 for all others. This arrangement accounts both for our stronger reliance on the asymptotic measures, and for our knowledge about the direction of the bias in the pre-step averages. In all cases this equation provided an exceptional fit, accounting for more than 99% of the variance in the data, regardless of the formula used to compute the coefficient of determination, R^2^ [22].

**Figure 3:**
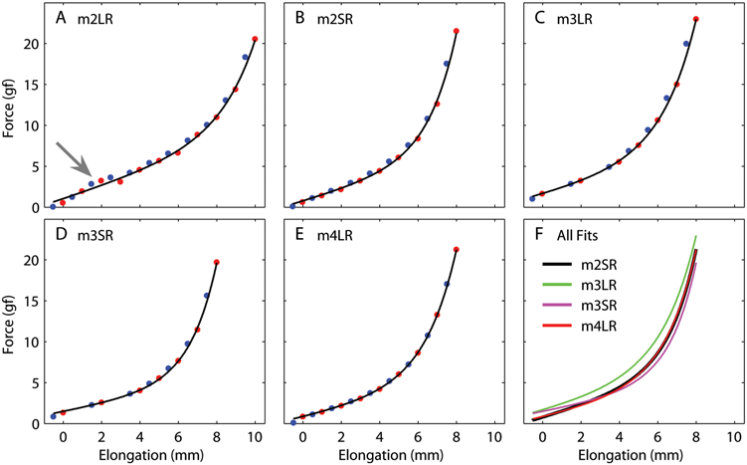
Passive force as a function of length in five eye muscles from three monkeys. A–E: Length-Tension curve for a single muscle. Blue points: average of the force measured during 200 ms before each step. Red points: estimate of the force at the final elongation after each step using the steady-state value from the spectral fit (see text). Black line: fit based on Eq. 1. F: Fits from panels B–E. The first muscle was excluded because the muscle was “cleaned” too extensively and it slipped around the globe (gray arrow in panel A). Muscles: lateral rectus (LR), superior rectus (SR).

We noted at recording time, and confirmed during the analysis, that the data from one of the muscles in the dataset was problematic. When we prepared the LR in our first monkey (Fig. 3A), we cleaned the orbital side too much. Lack of any connective tissues allowed the muscle to slip freely around the globe, so that it followed the shortest path from its origin to our apparatus. In spite of our best efforts to select an appropriate pulling direction, as soon as the force increased the muscle slipped around the globe (because the LR is the muscle that wraps around the globe the most, it is also the most sensitive to this problem). This can be easily seen in the data, as the slope dropped after about 3 mm (gray arrow Fig. 3A), and we had to elongate the muscle much more than in the other experiments to get to the steeper part of the curve. This again highlights how crucial it is to properly prepare the muscle in an *in vivo* experiment. The data acquired from this muscle was not subjected to any further analysis and discarded. The fitted parameters for the other four muscles (Fig. 3B–E) are listed in Table 1. In Fig. 3F we plot the length-tension curves from these four muscles together: they are surprisingly similar, even though the parameters listed in the table are not that close. That is because the parameters in Eq. 1 are not orthogonal, and thus can be traded off against each other without changing the fit dramatically.

**Table 1:** Length-Tension relationship parameters (Eq. 1).

	a	b	c	d
**m2SR**	0.687	0.0612	1.452	0.71
**m3LR**	0.602	0.1908	1.789	1.49
**m3SR**	0.457	0.0469	1.394	1.43
**m4LR**	0.519	0.1289	1.651	0.73

To estimate the stiffness of the muscles at short elongations (corresponding to small eye eccentricities), we also fitted a straight line to the portion of the curves up to elongations of 2 mm (note that we used the fitted equations, not the original data, as there were too few data points below that range). We obtained the following values: 0.78 (m2SR), 0.80 (m3LR), 0.53 (m3SR), and 0.67 (m4LR) gf/mm (corresponding to approximately 0.09 to 0.13 gf/° when expressed in terms of equivalent eye rotation).

It is important to note at this point that, while an attempt can be made to estimate the relative force of different muscles in the same animal from average geometrical/structural considerations, it is difficult to do so across monkeys. Thus, the best that we can expect to obtain from experiments such as ours, in which the number of muscles sampled is necessarily quite low, is an “average” description of muscle behavior, which can then be scaled for the various muscles within a model. To obtain such an average muscle model, we thus computed a population fit. Because the individual parameters in the model are not completely independent, looking at the values of the parameters in the individual fits was not particularly helpful. Instead, given how similar the curves for the muscles were, we fitted the equation directly to the pooled data points from all the muscles. We do realize that this is not a good idea in general, as we are pooling data across muscles and animals, but we felt that in our specific case it would be an efficient and effective way to obtain an expression that could be useful for modelers. The fit for this “average” muscle, this time done using unweighted least-square optimization, is:
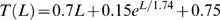
The stiffness at short elongations, in this case, is 0.85 gf/mm (or 0.14 gf/°), somewhat higher than that of any individual muscle. This is certainly due to the different fitting technique used for this curve, but the weighted least-squares optimization used above is not applicable in this context.

### Relaxation response

The relaxation response (i.e., the time course of the force decay following an elongation step) is sufficient to fully characterize a linear viscoelastic material. Not surprisingly then, its determination is the oldest and most extensively addressed problem in rheology. One would hope that such a long standing problem would have been solved by now, but it turns out to be an inverse problem very sensitive to noise (i.e., an ill-posed problem), and there is no “silver bullet” for its solution. In another paper we will compare several of the methods proposed over the years to fit the relaxation response, but here we will only describe the one that we found most convenient for the data presented herein.

Historically, viscoelastic models have been described using integer order differential equations. As the solution of such equations are exponential functions, it is only natural that the relaxation response has been modeled as a sum of exponentials:
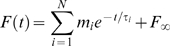
(2)The N moduli *m_i_* and time constants *τ_i_* define what is usually referred to as a line spectrum. Our goal is to find N and the values *m_i_* and *τ_i_* that, when plugged into Eq. 2, yield the best fit to the measured relaxation responses.

One way to tackle this problem is to arbitrarily impose N and the values for *τ_i_*, and then use an analytic least-squares error minimization method to find the optimal moduli *m_i_*. However, if the time constants are not far enough apart, this technique produces some negative moduli, which are obviously not acceptable. To overcome this problem, Emri and Tschoegl [23]–[26] developed a method to find a good fit (but not necessarily the best solution) without generating any negative moduli. With this method the user must still supply the time constants of interest, but the moduli are then computed using an iterative process that utilizes only non-overlapping data subsets for each *m_i_*. The algorithm is iterative and deterministic (i.e., the same result is obtained if run multiple times on the same data set), and very fast. Unfortunately, even with this method the choice of the time constants is critical, and the largest time constant is fit first and is thus privileged over the others. In addition to the time constants, the asymptotic force (*F_∞_*) must also be supplied, as the sum of decaying exponentials will necessarily converge to zero. Because we could not find established criteria to guide our choice of time constants and asymptotic force, we developed our own.

Emri and Tschoegl suggested simply using the value of the last data point as the asymptotic force, but that works only if the recording window is several times longer than the largest time constant. In our experiment that is definitely not the case, ruling out this approach. As an alternative, we used the following technique. First, we picked the highest time constant (40 s) that we felt we could estimate reliably given the duration of our recording window (30 s in m2 and m3, 45 s in m4); we used a single value for all muscles so that comparisons could be made more easily. Judging from experiments in other passive tissues, longer time constants most likely are in play, but cannot be reliably measured with our recording window. Next, we picked a fixed spacing (0.5 log_10_ units) for the time constants, and computed additional time constants down from the maximum to a lower bound of 1 ms (a limit imposed by the bandwidth of our equipment). We then used the Emri-Tschoegl (E-T) algorithm to compute the corresponding moduli for the best fit to one trace (starting from the end of the elongation). We did this several times, each time with a different asymptotic value (ranging from 0.75 to 0.99 times the final force). The value that yielded the best fit was selected as the asymptotic force, F_∞_, and was then used to compute the length-tension curve in Eq. 1, as described above.

**Figure 4:**
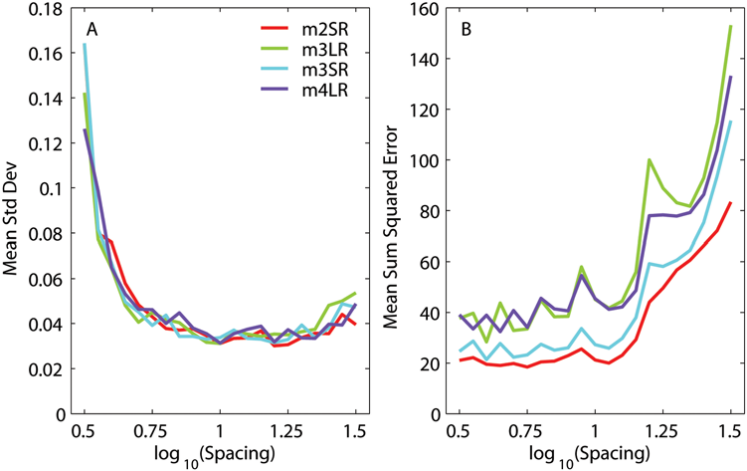
Relationship between fit quality and line spectrum spacing. A: The E-T algorithm was run on several simulated relaxation responses, each obtained by adding noise to the fit to an actual relaxation response (see text). As the spacing between spectrum lines (abscissa) decreases, the variability in the moduli of the fits to the simulated responses increases, because moduli for neighboring time constants are traded off against each other. As the spacing is increased this trade off is not possible anymore, and the variability in the fit reflects only the noise in the fitted responses. For each spacing, we computed, for each time constant, the standard deviation of the moduli over the set of fits to the simulated responses. We plot the mean of this measure over all the time constants against the spacing, separately for each muscle. B: As the spacing between spectrum lines (abscissa) increases, the fit deteriorates. For a given spacing, we computed the sum squared error for the fit to each simulated response. We plot the mean of this measure as a function of spacing, separately for each muscle.

Once the asymptotic force was obtained, we subtracted the corresponding fit (Eq. 2) from the force trace, and analyzed the statistics of the residual noise (after cubic detrending, if necessary). We then used an Ornstein-Uhlenbeck process [27] to generate noise with the same variance and similar auto-correlation function, added this new noise to the fit previously obtained (adding back the detrending curve when necessary), thus generating a synthetic force measurement. We repeated this step several times (10 to 50), thus producing a family of synthetic force traces carrying the same signal but different noise instances. We then ran the E-T algorithm on each of these traces, and we did so for many different time constant spacings (from 0.5 to 1.5 log_10_ units). We found that when the spacing between time constants was too small, on different fits neighboring moduli were traded off against each other, so that when one was high the other was low, and *vice versa*. Thus, the standard deviation of the moduli for any one time constant, across runs, was quite large. When the spacing was large enough, this phenomenon did not occur, and all that was the left were the small changes expected given the noise level. We found that, with our noise level, this occurred for a spacing between 0.7 and 0.8 decades (Fig. 4A). On the other hand, as the spectrum lines become too far apart, the quality of the fit, evaluated computing the mean SSE across the set of synthetic force traces, deteriorates. At our noise levels this occurred above one decade (Fig. 4B). Accordingly, any spacing between 0.75 and one decade could be used. In all cases we used a spacing of 0.75 decades, corresponding to 7 spectrum lines in the range 1 ms to 40 s. We settled on the lower end of the spacing spectrum because it enabled us to get better fits during the elongation phase (see below). At this spacing level the analytic least-squares method was still generating one or more negative moduli, and thus was not a viable alternative to the E-T algorithm.

**Figure 5:**
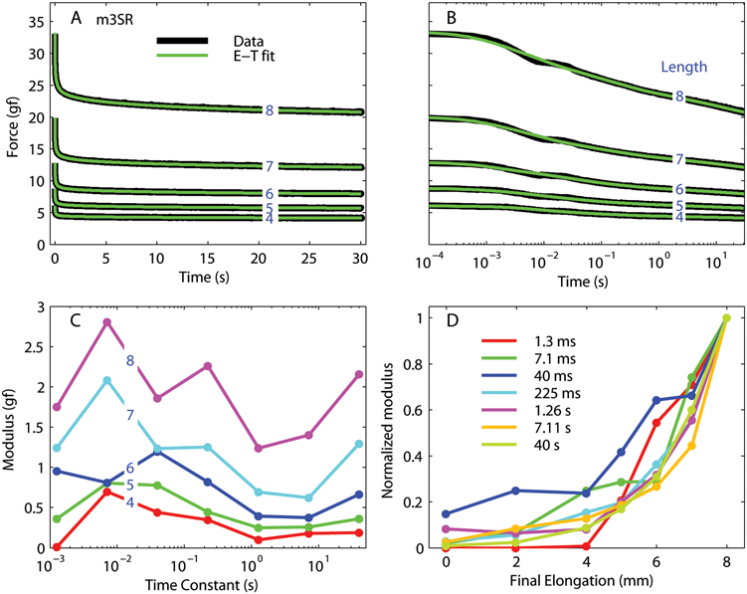
Relaxation responses from the superior rectus in m3. A: Data (black) and fits (green) for five different steps (blue numbers are the final length in mm). The steps at the smallest final length are not plotted for clarity, but the fits were just as good. B: Same as A, but using a logarithmically spaced abscissa to improve visualization of the force at short times. C: Moduli associated with each time constant in the fit; one line for each final length. D: Normalized moduli as a function of muscle length after the step; one line for each time constant. Note that in this panel the shortest lengths are also represented.

Once we had found the spacing for the spectrum lines and, for each muscle, the length-tension curve, we computed the line spectrum from each quick-step trace. This algorithm yielded excellent fits to our post-elongation decays, with a median r^2^ value of 0.9972 (ranging between 0.9783 and 0.9995, excluding the fits at the two shortest lengths, where the force was small compared to the noise). In Fig. 5 we show the fit for some of the steps recorded from the superior rectus muscle of m3. In panel A the data and the fits are shown with a linear scale, whereas in panel B the same data and fits are shown with a logarithmic scale (to improve visualization at short times). The fits are excellent at all lengths and over the entire duration of the experiment, with the exception of a small disturbance around 10 ms (most likely an artifact due to a small transient overshoot of the final length). In panel C we plot the moduli for the fits plotted in A and B. Seven time constants were used, spaced by 0.75 decades (1.3 ms, 7.1 ms, 40 ms, 225 ms, 1.26 s, 7.11 s, and 40 s). While an overall trend is clear across lengths, this is not strict, especially for the small time constants. In panel D we plot, for each time constant, the moduli as a function of muscle length, normalized to the peak value. For most time constants, the moduli increase “exponentially” with length, even though there are some obvious differences between the various curves.

**Figure 6:**
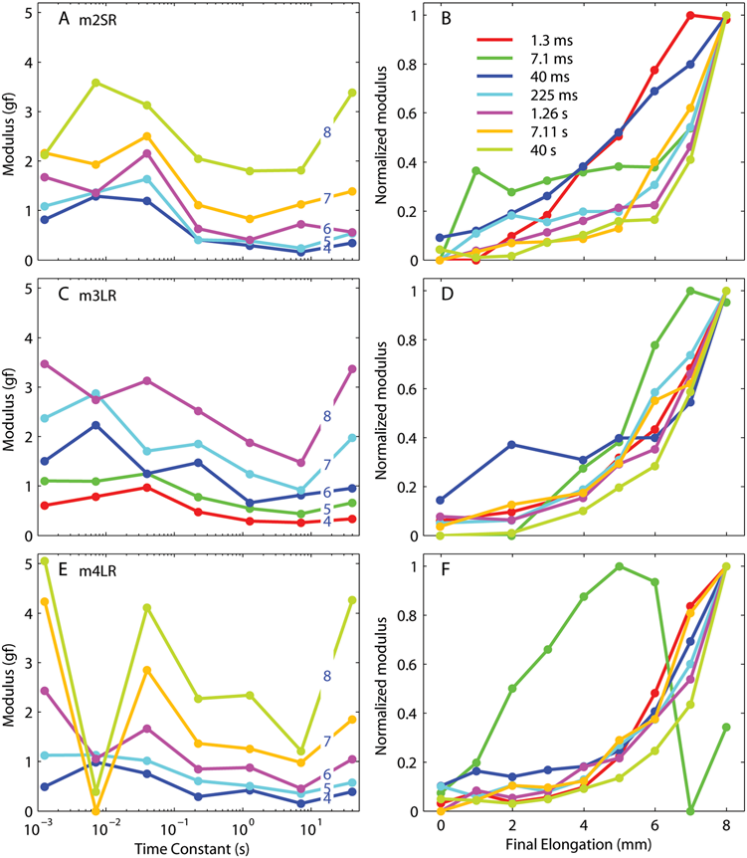
Relaxation responses from the other three muscles tested. One row per muscle. The panels on the left are the same as Fig. 5C, whereas the panels on the right are the same as Fig. 5D. Note the variability of the moduli both as a function of final length and time constants.

In Fig. 6 we plot the spectra (as in panels C and D in Fig. 5) for the other three muscles tested. The overall trend is the same, but again there is quite a lot of variability. The moduli associated with the 7 ms time constant are particularly idiosyncratic, especially in m4 but to a lesser extent also in the other monkeys. We do not have a good explanation for this discrepancy, and unfortunately studies of other passive tissues do not usually investigate these short time scales. It is hard to rule out that measurement artifacts, such as the propagation of inertial waves [28], are responsible. One could suspect that the somewhat higher compliance of the method used to connect this muscle to the apparatus might have played a role, but then the effect should have been even more pronounced on the 1 ms time constant. Given the high values of the 1 ms modulus relative to the 7 ms modulus in this muscle (a more compliant connection would be expected to produce the opposite result), the most likely explanation is that the moduli for the two time constants have been traded off versus each other (i.e., probably six time constants should have been used for this muscle, but to make comparisons across muscles we wanted to use the same fitting equation for all muscles). As a general rule, the moduli for time constants over 20 ms increased monotonically and “exponentially” with length, whereas the moduli for the two shortest time constants are more variable and less stereotypical. The values of the moduli for the individual muscles are listed in Tables 2–5.

**Table 2:** Relaxation spectrum in m2SR (0.5 mm steps from different initial lengths).

	1.3 ms	7.1 ms	40 ms	225 ms	1.26 s	7.11 s	40 s
**0**	0.000	0.000	0.286	0.000	0.000	0.000	0.143
**1**	0.000	1.306	0.372	0.223	0.070	0.058	0.036
**2**	0.211	0.995	0.595	0.371	0.130	0.126	0.056
**3**	0.397	1.165	0.820	0.318	0.205	0.134	0.243
**4**	0.817	1.286	1.197	0.405	0.287	0.157	0.347
**5**	1.090	1.369	1.633	0.404	0.383	0.232	0.539
**6**	1.677	1.358	2.159	0.629	0.404	0.727	0.560
**7**	2.160	1.930	2.501	1.111	0.834	1.126	1.389
**8**	2.123	3.585	3.131	2.047	1.798	1.811	3.386

**Table 3 pone-0004850-t003:** Relaxation spectrum in m3LR (0.5 mm steps from different initial lengths).

	1.3 ms	7.1 ms	40 ms	225 ms	1.26 s	7.11 s	40 s
**0**	0.208	0.000	0.452	0.128	0.146	0.054	0.000
**2**	0.335	0.000	1.161	0.156	0.120	0.185	0.035
**4**	0.605	0.787	0.967	0.474	0.289	0.258	0.337
**5**	1.104	1.098	1.246	0.778	0.548	0.434	0.662
**6**	1.505	2.235	1.250	1.472	0.663	0.813	0.955
**7**	2.371	2.877	1.706	1.856	1.239	0.915	1.977
**8**	3.469	2.741	3.130	2.518	1.878	1.476	3.369

**Table 4 pone-0004850-t004:** Relaxation spectrum in m3SR (0.5 mm steps from different initial lengths).

	1.3 ms	7.1 ms	40 ms	225 ms	1.26 s	7.11 s	40 s
**0**	0.000	0.059	0.274	0.065	0.102	0.035	0.020
**2**	0.000	0.188	0.463	0.129	0.080	0.119	0.053
**4**	0.012	0.697	0.442	0.350	0.101	0.179	0.190
**5**	0.361	0.805	0.775	0.444	0.248	0.261	0.364
**6**	0.953	0.808	1.197	0.819	0.395	0.374	0.667
**7**	1.242	2.083	1.233	1.251	0.691	0.623	1.294
**8**	1.752	2.806	1.861	2.258	1.241	1.402	2.159

**Table 5:** Relaxation spectrum in m4LR (0.5 mm steps from different initial lengths).

	1.3 ms	7.1 ms	40 ms	225 ms	1.26 s	7.11 s	40 s
**0**	0.163	0.083	0.419	0.230	0.000	0.000	0.207
**1**	0.377	0.223	0.671	0.133	0.194	0.054	0.184
**2**	0.179	0.566	0.578	0.238	0.126	0.126	0.130
**3**	0.283	0.746	0.688	0.179	0.187	0.117	0.211
**4**	0.494	0.990	0.751	0.291	0.424	0.149	0.391
**5**	1.126	1.131	1.017	0.611	0.507	0.351	0.579
**6**	2.434	1.058	1.671	0.847	0.879	0.456	1.052
**7**	4.233	0.000	2.850	1.363	1.260	0.981	1.853
**8**	5.058	0.387	4.112	2.268	2.340	1.213	4.266

### Force during the step

So far we have described what happens at equilibrium, and how the muscle force relaxes after a step. To conclude our analysis of the step response of passive muscles, we need to describe what happens *during* the elongation. These data can be a significant source of information, but they are often neglected, both in muscle physiology and in rheological studies of natural or man-made materials. As far as we know, Ford and colleagues [29] were the first to propose that imposing a very brief constant-velocity stretch (i.e., a ramp change in length) is an ideal method to study the viscoelastic behavior of muscles. Subsequently, Bagni and colleagues [30], [31] used this paradigm on frog skeletal muscle fibers, and Mutungi and Ranatunga [32], [33] used it to study rat skeletal muscle fibers. When we planned our experiments we thus made sure that our steps were generated in this manner, i.e., with very fast acceleration and deceleration phases, and a constant velocity for most of the step duration. For example, for a step with a peak speed of 80 mm/s, the peak speed was reached in 1.5 ms, it was maintained for 5 ms, and the deceleration phase lasted another 1.5 ms, for a total of 8 ms.

**Figure 7:**
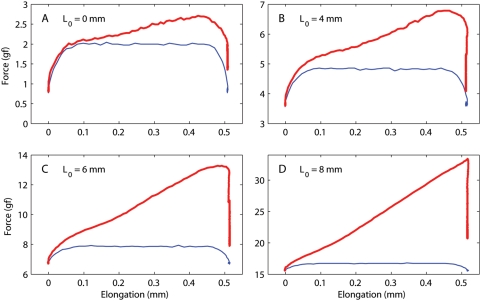
Force measured during a 0.5 mm step. All data shown are from the superior rectus (SR) of the second monkey (m3). Each panel shows force (red) and normalized speed (blue) as a function of change in muscle length, for different initial lengths spanning the entire range tested. (The speed trace is used only to indicate how it varies during the elongation; its magnitude has no meaning).

To gain the maximum insight, it is useful to plot the force not as a function of time, but rather as a function of muscle elongation. Of course, by doing so the post-step relaxation collapses to a vertical line. In Fig. 7 we plot, as a function of elongation, both the force (red traces) and the speed (blue traces, multiplied by 0.015 for clarity; the peak speed was 80 mm/s for these steps) for steps executed at different muscle lengths (L_0_). At short lengths (panel A) it is obvious that initially the force increases linearly with speed, indicating a purely viscous process, or at least a process with an extremely short time constant (no more than 0.2 ms, given that the peak speed is reached in 1.5 ms). If we subtract off this viscous contribution, what is left is the typical response of a linear viscoelastic system, and it is thus compatible with the relaxation response that we just described. This same explanation works fairly well also at the next length (panel B, note change in scale), but it breaks down for the last two (panels C and D). Note that, in these latter two cases, there is an upward inflection in the force trace long after the speed has become stable. The upward inflection is due to the quickly increasing stiffness as the muscle is stretched; this type of behavior cannot be produced by a linear system (i.e., a system with constant stiffness). This is compatible with the moduli shown in Fig. 5D: at short lengths, the moduli do not vary by much over the 0.5 mm elongation range covered by the steps, but as the length increases they change considerably even over such short elongations. Only a nonlinear system can generate this type of behavior.

### Variation across muscles

**Figure 8:**
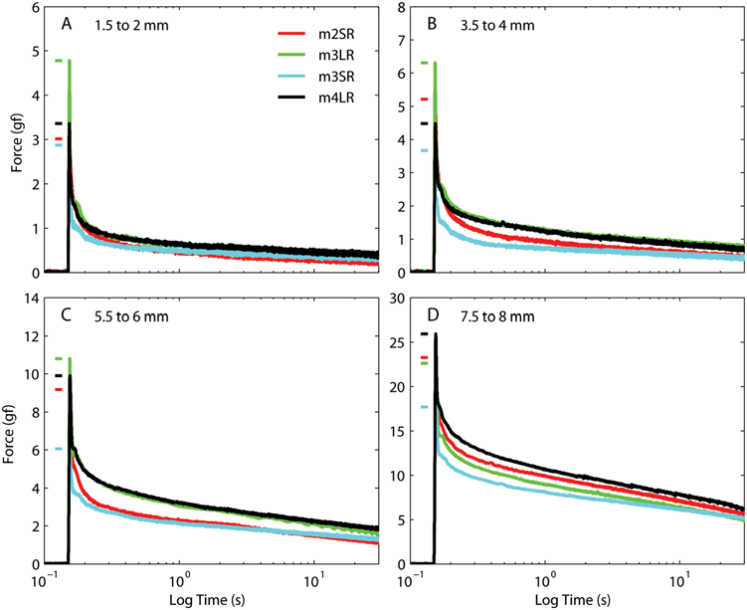
Force induced by 0.5 mm steps in different muscles. The initial length is different in each panel. The short horizontal bars on the left of each trace indicate the maximum force for that muscle. The initial static force, slightly different for each muscle (Fig. 3), has been subtracted from each trace.

Because it is quite difficult to estimate the amount of variability observed across muscles for the numerical fits reported above, in Fig. 8 we plot, for all muscles and separately at different lengths, the viscoelastic forces induced by elongation steps of identical amplitude and speed. To better focus on the viscoelastic force, we subtracted from the traces the initial (static) force, which, as shown in Fig. 3, is slightly different for each muscle. The final (asymptotic) force is also different in different muscles, but we did not make any adjustment for that. Note that there is a fair amount of variation across muscles, but the relative forces do not simply scale at different lengths. So, for example, the lateral rectus in m3 (green traces) exhibits the largest maximum force (indicated for clarity by short horizontal bars) in the first three steps (panels A–C), but not in the last. The temporal evolution of the force decay is also somewhat variable: for example, in panel D higher peak forces result in higher forces throughout the decay phase, but that is not true in panel B. All in all, this shows that while the muscles certainly behave in qualitatively similar ways both statically and dynamically, they also exhibit some substantial quantitative differences.

## Discussion

### A graphical representation

**Figure 9:**
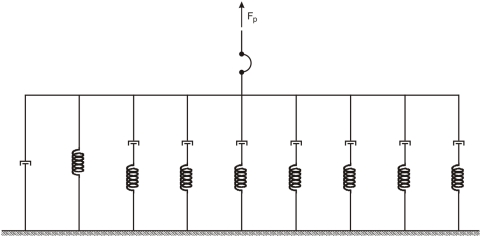
A series-parallel representation compatible with the mechanical properties of passive muscles reported in this paper. This must not be interpreted as a model, because it has no predictive value. Rather, it is a didactically useful cartoon, a way of graphically summarizing our findings. Symbols: the arc is a limp leash (muscles pull, but don't push), a spring (stiffness), and a dashpot damper (viscosity).

We noted in the introduction that Collins modeled the passive muscle with an elastic element in series with a Voigt element. These types of representations, referred to as *series-parallel mechanical models*, have been used extensively to describe linear viscoelastic behavior [34]. They are less useful to represent nonlinear models, but they can nonetheless be didactically useful. In this vein, and with these caveats, we could then say that, from the data presented so far, a better representation for passive extraocular muscles (Fig. 9) is obtained by connecting in parallel a spring, a damping element, and seven Maxwell elements (a viscous and an elastic element in series). Each Maxwell element accounts for one of the relaxation processes, the spring accounts for the static length-tension relationship, and the damper accounts for the viscous force that can be observed during the step. Again, it cannot be stressed enough that this is just a representation, a cartoon if you wish, and must not be interpreted as a model. There are infinite other representations that could fit the data presented, and they would all behave very differently when tested on other elongation histories.

### The length-tension relationship

The relationship between the static (i.e., steady-state) tension exerted at the tendon of a resting eye muscle and its length has been measured experimentally in cats (e.g., [4], [5]), in monkeys [8], and in humans (e.g., [5], [11], [12], [35]). As mentioned in the Introduction, the different measurements do not agree quantitatively. Nonetheless, there is a qualitative consensus that the relationship between passive force and muscle length is highly nonlinear, and that the force increases faster and faster as the muscle is stretched. Here we confirmed these findings, and quantified this relationship in monkey EOMs. We found that expressions used previously to fit this curve in eye [5], [20] and skeletal [21] muscles were not appropriate for our data. The expression we proposed has one extra degree of freedom, but we showed in the Introduction that it is in fact appropriate even for the older datasets. In addition, it captures well our finding that the force increases approximately linearly with length over the first 3 mm (corresponding to 18° of eye rotation). It is important to stress that this last observation does *not* imply that the muscle behaves linearly within this range, because linearity requires both scaling and superposition, and there is no experimental evidence that the latter holds. Unlike the previous experiments listed in the Introduction, we found that the static relationship is remarkably consistent across muscles and monkeys (Fig. 3F). Larger variations might have been observed in the oblique muscles, but it is technically very difficult to record these muscles *in vivo*.

It should be remarked that our findings do *not* match quantitatively the curve reported by Fuchs and Luschei [8], the only other recording in monkeys (see Introduction). More precisely, over the first 2–3 mm of elongation they report a much smaller stiffness, estimated by Sklavos and colleagues [36] at 0.03 gf/°, three to four times smaller than what we report here. Also, over this elongation range the forces they report were not even monotonic. There are several reasons that could explain the discrepancy; first of all, it is not clear exactly how those authors measured the passive force (most of their experiment was devoted to the measurement of active forces). The history of elongation leading up to the measurement of the passive force is of course very important, and it was very tightly controlled in our experiments. Long and consistent pre-measurement delays, which we used throughout, are unlikely to have been part of their protocol. Second, their surgical procedure was much more invasive than ours (the lateral bone of the orbit was removed). Third, their force sensor was a lot less accurate than ours (5% linearity vs. 0.2% linearity). Finally, if one were to take at face value their measurements of the passive force at large lengths and tetanic force at small lengths, the oculomotor range of the monkey would be limited to less than 35°, which is much less than the actual range (45°). Unfortunately, data from only one muscle was shown (five lateral recti were tested), and no analytical fits were provided. For all these reasons, we are confident that our measurements provide a superior estimate of the passive forces in monkey extraocular muscles.

### Dynamic forces

All the passive biological tissues ever studied, such as tendons, ligaments, veins, arteries, cartilage, cardiac and skeletal muscle, are characterized by a relaxation response that exhibits a wide range of time scales, from 1 ms all the way up to 1 hour (almost 7 orders of magnitude). As noted in the Introduction, Collins [5] was the only one to have attempted to measure the dynamic forces exerted by a passive eye muscle (cat lateral rectus). His proposed model (a single relaxation process with a 100 ms time constant) would imply that passive eye muscles have little in common with any other passive biological tissue studied. It has been known for a while that extraocular muscles exhibit several distinctive structural and functional characteristics [37], but we were highly suspicious that these could lead to such a drastic difference in their mechanical properties. This study demonstrates that passive eye muscles are in fact not qualitatively different from other biological passive tissues. We found that a set of dynamic processes could be inferred, with time constants ranging from 1 ms to at least 40 s. The moduli associated with these time constants were comparable, so that it cannot be said that any one time constant dominates.

We introduced here an algorithm that permits objective determination of how far apart the spectrum lines must be to avoid over-fitting or under-fitting the response. While we are aware that the most likely scenario is that there is actually a continuous range of processes, each with a different time constant, the noise inevitably present in any recording makes it impossible to do better than the line spectrum presented here [38]. Similarly, we have no reasons to exclude that much longer time constants (e.g., 10 minutes) could be observed, as they are routinely reported in collagen (e.g., [39]) and skeletal muscles (e.g., [40]). However, the *in vivo* conditions under which we performed our experiments imposed a trade-off between observation time and number of experiments that could be performed. We believe that our choice was justified given the broad range of questions we were interested in addressing (described in the following papers in this series).

### Origin of the passive force

In the early days of muscle research, it was assumed that the passive force is generated mainly by the connective tissues that support the myoplasm (epimysium, perimysium, and endomysium), and not by the myoplasmic proteins themselves [41], [42]. However, it was shown later that, at least in amphibian skeletal muscles, the myofibrils themselves must be generating most of this force, at least at small elongations [40]. Careful study of individual fibers revealed that the giant protein titin is the main contributor of this force [43], [44].

One might then be tempted to think that this issue is settled, and it might be so for skeletal muscles, but extraocular muscles have an important peculiarity. In skeletal muscles, both at the single fiber level and at the whole muscle level, the passive force becomes significant only after the active force has peaked (i.e., at long lengths) [41], so that the total force exhibits a region of “negative stiffness”. In extraocular muscles, at least at the whole muscle level, this does not happen, because the passive force starts increasing before (i.e., at shorter lengths) the active force peaks, so that when the latter decays the passive force picks up the slack. Accordingly, the stiffness for the total force is always positive [4]. To the best of our knowledge, the rationale for this difference has not been studied, but it is reasonable to hypothesize that it has to do with the considerably larger elongation range (relative to the muscle resting length) over which extraocular muscles have to operate. In skeletal muscles the operating range is restricted to lengths shorter than that for which the active force peaks [45], and so the negative stiffness region is never reached. However, the ratio between the diameter of the eye, i.e., the joint that eye muscles rotate, and muscle length at rest is around one in monkeys, much larger than for any skeletal muscle, so that extraocular muscles must operate over a much larger length range. How this specialization has been achieved is however not known. Importantly, it is not even known whether this holds at the single fiber level. The definitive experiment to elucidate this issue requires the measurement of active and passive properties in a skinned extraocular muscle fiber. Comparing the force in passive and chemically activated fibers would explain whether this difference is due to a shorter titin protein in extraocular muscles (in which case the single fiber total stiffness would also be always positive), or whether it is in fact the collagen surrounding the fibers that provides most of the passive force in whole extraocular muscle (in which case the single fiber total stiffness would be similar to that observed in skeletal muscles).

The data presented here cannot resolve this issues, but it can be used to make some inferences. The length-tension relationship has been measured countless times in single fibers, and even individual sarcomeres, in skeletal muscles (e.g., [46], [47]). However, when single fibers are studied, the length-stiffness relationship (i.e., the slope of the length-tension curve) increases with length up to a certain point, and then saturates (i.e., it looks sigmoidal, e.g., [46], their Fig. 6). Interestingly, studies of single titin filaments reveal elastic properties virtually identical to those of an intact muscle fiber [47]. Furthermore, a careful study of cat skeletal muscles led Brown and colleagues [21] to propose an equation for the the passive properties of whole muscles that is also characterized by stiffness saturation.

If we look closely at our data it appears clear that the stiffness of the muscles we studied does not saturate. This can be appreciated both from the analytical expression of the stiffness
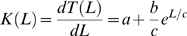
and from the actual data (note in Fig. 3 how the distance between two successive red dots always increases with elongation, with no sign of saturation). This suggests that titin might not be the source of the passive force we measured, which could instead be generated mostly by the collagen network that supports the myoplasm. It is true that the stiffness of individual collagen fibers also saturates [48], but it is conceivable that inhomogeneity in the length of individual fibrils within the collagen network, presumably much larger than sarcomere inhomogeneity in the myoplasmic network [45], might play a significant role. Of course it is also possible that the relative contribution of these two sources changes with length, as shown in cardiac muscle [49].

### Implications for models of the eye plant

As we have shown in this paper, the dynamic properties of passive extraocular muscles are very different from what has been long assumed. It is then not surprising that, based on those assumptions, dynamic deficits (especially post-saccadic drifts) associated with paralytic strabismus appear puzzling. Our study also highlights the fact that, in spite of over forty years of intense study of the oculomotor system, our understanding of even its most basic physiology is still quite limited. Worse, a lot of what we think we know might, in fact, turn out to be incorrect. For example, besides what we have shown here, very little is known about the properties of naturally innervated muscles. There are countless studies that describe how the force changes when either the muscle length or the (artificial) innervation is changed while the other is kept constant, but independent force and length changes never happen in natural conditions. Similarly, the interactions between active and passive properties are routinely ignored. It has been known for almost sixty years [50] that the relaxation spectrum is much narrower for a tetanically innervated muscle than for a passive muscle, indicating a highly nonlinear interaction. However, most models either ignore the passive properties (which might be acceptable in some cases for skeletal muscles, but never for extraocular muscles), or it is simply assumed that passive and active force are independent and just sum. Some of the dynamic deficits associated with muscle paralysis (e.g., hysteresis) might then be due to the wider relaxation spectrum of passive muscles. Similar criticisms can be applied to models of individual sarcomeres, as it has been shown that the crossbridge and non-crossbridge force components also interact, and do not simply sum [51], [52].

Only models that more realistically reproduce the complex dynamic behavior of muscles will have any hope of capturing the type of subtle deficits often observed in the clinic, which might very well have a high, but as yet untapped, diagnostic value. In a subsequent paper in this series we will describe a model that does a fair job of reproducing the passive properties of extraocular muscles, but a lot more remains to be done, both on the experimental and on the theoretical side.

## References

[1] Shan X, Tian J, Ying HS, Quaia C, Optican LM (2007). Acute superior oblique palsy in monkeys: I. Changes in static eye alignment.. Invest Ophthalmol Vis Sci.

[2] Shan X, Ying HS, Tian J, Quaia C, Walker MF (2007). Acute superior oblique palsy in monkeys: II. Changes in dynamic properties during vertical saccades.. Invest Ophthalmol Vis Sci.

[3] Quaia C, Shan X, Tian J, Ying H, Optican LM (2008). Acute superior oblique palsy in the monkey: effects of viewing conditions on ocular alignment and modelling of the ocular motor plant.. Prog Brain Res.

[4] Robinson DA (1964). The mechanics of human saccadic eye movements.. J Physiol.

[5] Collins CC, Bach-y-Rita P, Collins CC, Hyde JE (1971). Orbital mechanics.. The control of eye movements.

[6] Barmack NH, Bell CC, Rence BG (1971). Tension and rate of tension development during isometric responses of extraocular muscle.. J Neurophysiol.

[7] Barmack NH (1976). Measurements of stiffness of extraocular muscles of the rabbit.. J Neurophysiol.

[8] Fuchs AF, Luschei ES (1971). Development of isometric tension in simian extraocular muscle.. J Physiol.

[9] Stone SL, Thomas JG, Zakian V (1965). The passive rotatory characteristics of the dog's eye and its attachments.. J Physiol.

[10] Breinin GM (1962). The electrophysiology of extraocular muscle.

[11] Robinson DA, O'Meara DM, Scott AB, Collins CC (1969). Mechanical components of human eye movements.. J Appl Physiol.

[12] Collins CC, O'Meara D, Scott AB (1975). Muscle tension during unrestrained human eye movements.. J Physiol.

[13] Scott AB, Bach-y-Rita P, Collins CC, Hyde JE (1971). Extraocular muscle forces in strabismus.. The control of eye movements.

[14] Robinson DA (1981). Models of the mechanics of eye movements.. Models of oculomotor behavior and control.

[15] Bach-y-Rita P, Ito F (1966). In vivo studies on fast and slow muscle fibers in cat extraocular muscles.. J Gen Physiol.

[16] Croes SA, von Bartheld CS (2007). Measurement of contractile force of skeletal and extraocular muscles: effects of blood supply, muscle size and in situ or in vitro preparation.. J Neurosci Methods.

[17] Miller JM, Robinson DA (1984). A model of the mechanics of binocular alignment.. Comput Biomed Res.

[18] Hakim OM, Gaber El-Hag Y, Maher H (2008). Persistence of eye movement following disinsertion of extraocular muscle.. J AAPOS.

[19] Noll W, Coleman BD (1961). Foundations of linear viscoelasticity.. Rev Mod Phys.

[20] Robinson DA (1975). A quantitative analysis of extraocular muscle cooperation and squint.. Invest Ophthalmol.

[21] Brown IE, Scott SH, Loeb GE (1996). Mechanics of feline soleus: II. Design and validation of a mathematical model.. J Muscle Res Cell Motil.

[22] Willett JB, Singer JD (1988). Another cautionary note about r2: its use in weighted least-squares regression analysis.. Am Stat.

[23] Emri I, Tschoegl NW (1994). Generating line spectra from experimental responses. 4. Application to experimental-data.. Rheol Acta.

[24] Emri I, Tschoegl NW (1993). Generating line spectra from experimental responses. 1. Relaxation modulus and creep compliance.. Rheol Acta.

[25] Tschoegl NW, Emri I (1993). Generating line spectra from experimental responses. 2. Storage and loss functions.. Rheol Acta.

[26] Emri I, Tschoegl NW (1995). Determination of mechanical spectra from experimental responses.. Int J Solids Struct.

[27] Gardiner CW (2004). Handbook of stochastic methods.

[28] Nekouzadeh A, Genin GM, Bayly PV, Elson EL (2005). Wave motion in relaxation-testing of nonlinear elastic media.. Proc Roy Soc A-Math Phy.

[29] Ford LE, Huxley AF, Simmons RM (1977). Tension responses to sudden length change in stimulated frog muscle fibres near slack length.. J Physiol.

[30] Bagni MA, Cecchi G, Colomo F, Garzella P (1992). Are weakly binding bridges present in resting intact muscle fibers?.. Biophys J.

[31] Bagni MA, Cecchi G, Colomo F, Garzella P (1995). Absence of mechanical evidence for attached weakly binding cross-bridges in frog relaxed muscle fibres.. J Physiol.

[32] Mutungi G, Ranatunga KW (1996). The visco-elasticity of resting intact mammalian (rat) fast muscle fibres.. J Muscle Res Cell Motil.

[33] Mutungi G, Ranatunga KW (1996). The viscous, viscoelastic and elastic characteristics of resting fast and slow mammalian (rat) muscle fibres.. J Physiol.

[34] Tschoegl NW (1989). The phenomenological theory of linear viscoelastic behavior.

[35] Collins CC (1975). The human oculomotor control system.. Basic mechanisms of ocular motility and their clinical implications.

[36] Sklavos S, Porrill J, Kaneko CRS, Dean P (2005). Evidence for wide range of time scales in oculomotor plant dynamics: implications for models of eye-movement control.. Vision Res.

[37] Porter JD, Baker RS (1996). Muscles of a different ‘color’: the unusual properties of the extraocular muscles may predispose or protect them in neurogenic and myogenic disease.. Neurology.

[38] Puso MA, Weiss JA (1998). Finite element implementation of anisotropic quasi-linear viscoelasticity using a discrete spectrum approximation.. J Biomech Eng - T ASME.

[39] Pryse KM, Nekouzadeh A, Genin GM, Elson EL, Zahalak GI (2003). Incremental mechanics of collagen gels: new experiments and a new viscoelastic model.. Ann Biomed Eng.

[40] Magid A, Law DJ (1985). Myofibrils bear most of the resting tension in frog skeletal muscle.. Science.

[41] Ramsey RW, Street SF (1940). The isometric length-tension diagram of isolated skeletal muscle fibers of the frog.. J Cell Compar Physiol.

[42] Hill AV (1950). Mechanics of the contractile element of muscle.. Nature.

[43] Horowits R, Kempner ES, Bisher ME, Podolsky RJ (1986). A physiological role for titin and nebulin in skeletal muscle.. Nature.

[44] Wang K (1996). Titin/connectin and nebulin: giant protein rulers of muscle structure and function.. Adv Biophys.

[45] Goulding D, Bullard B, Gautel M (1997). A survey of in situ sarcomere extension in mouse skeletal muscle.. J Muscle Res Cell Motil.

[46] Horowits R, Podolsky RJ (1987). The positional stability of thick filaments in activated skeletal muscle depends on sarcomere length: evidence for the role of titin filaments.. J Cell Biol.

[47] Wang K, McCarter R, Wright J, Beverly J, Ramirez-Mitchell R (1991). Regulation of skeletal muscle stiffness and elasticity by titin isoforms: a test of the segmental extension model of resting tension.. Proc Natl Acad Sci U S A.

[48] Viidik A, Viidik A, Vuust J (1980). Mechanical properties of parallel-fibred collagenous tissues.. Biology of collagen.

[49] Linke WA, Popov VI, Pollack GH (1994). Passive and active tension in single cardiac myofibrils.. Biophys J.

[50] Buchthal F, Kaiser E (1951). The rheology of the cross striated muscle fiber, with particular reference to isotonic conditions.. Dan Biol Medd.

[51] Pinniger GJ, Ranatunga KW, Offer GW (2006). Crossbridge and non-crossbridge contributions to tension in lengthening rat muscle: force-induced reversal of the power stroke.. J Physiol.

[52] Telley IA, Denoth J (2007). Sarcomere dynamics during muscular contraction and their implications to muscle function.. J Muscle Res Cell Motil.

